# Health Care Professionals' Perception of Stress During COVID-19 Pandemic in Iran: A Qualitative Study

**DOI:** 10.3389/fpsyt.2021.804637

**Published:** 2022-02-01

**Authors:** Ashraf Rouhbakhsh, Rahim Badrfam, Ali-Akbar Nejatisafa, Marzieh Soori, Sayedeh Elham Sharafi, Farnaz Etesam, Nazila Shahmansouri, Mohammad Arbabi, Ahmad Ali Noorbala

**Affiliations:** ^1^Department of Psychiatry, Psychosomatic Research Center, Tehran University of Medical Sciences, Tehran, Iran; ^2^Research Center for Cognitive and Behavioral Sciences, Tehran University of Medical Sciences, Tehran, Iran; ^3^Department of Psychiatry, Psychosomatic Research Center, Tehran University of Medical Sciences, Tehran, Iran

**Keywords:** COVID-19, health personnel, mental health, stress, qualitative study

## Abstract

**Background:**

The health care professionals have a unique role in controlling the pandemic of COVID-19 and decreasing its mortality and morbidity. The burden of care and psychological impact of working in this circumstance can be unfavorable for many caregivers. In this qualitative study, the health care professionals' perception of stress during COVID-19 pandemic in Iran was assessed and several implications were proposed.

**Materials and Methods:**

The participants were selected among staff who were providing medical services to patients with COVID-19 infection at the largest teaching hospital in Iran. Quota sampling was used to include physicians, nurses, and other paramedics. The grounded theory was selected to develop interview questions. Moreover, the thematic approach was applied to analyze the data content and data analysis was performed based on open and axial coding following the implementation of codes in MAQDA software.

**Results:**

A wide range of psychological reactions including anxiety, feelings of guilt, depression, and anger were detected in the staff. Uncertainty accompanied by the pandemic of COVID-19 and shortcomings in preparation for crisis management were recognized as the two main sources of stress among health care professionals.

**Conclusion:**

Based on the findings of the study, it is important to identify and evaluate the mental health needs of healthcare professionals. To reduce stress among health staff at COVID-19 care centers, it seems that the optimal strategy is simultaneous improvement in equipment and crisis management.

## Introduction

As of today, we have witnessed the spread of an invisible enemy which was envisaged as pneumonia of an unknown origin in late December 2019. The first reported cases of the disease and consequently the outbreak of the disease worldwide made the World Health Organization (WHO) determined to call it a pandemic. The disease was named COVID-19 and so far (14 November 2021), more than 252 million people have been infected and more than 5 million have died. Iran is among the countries with the highest number of people affected and the highest number of deaths due to COVID-19 in the Middle East (1).

Working in this crisis condition like the previous epidemics in the world (2) or natural disasters (3) put a heavy burden on the physicians, nurses, and other health care staff in the referral hospitals (4). The staff are directly involved with the virus and as a result, they should encounter the risk of transmitting the disease to themselves or their families. The other problems include mandatory quarantine or relative isolation, serious limitations in personal protection equipment (PPE), and minimum rest breaks (5, 6). Such problems and challenges result in psychological stress, followed by possible psychiatric manifestations, including anxiety, depression, and stigma (7–9). The staff's mental health problem is a critical issue since decreased care for patients and increased medical errors may be the adverse outcomes (10). Identifying these obstacles can be the first step in addressing and establishing mental health care for the staff (11). In dealing with such issues, regional and cultural features as a determining factor should be taken into account (12). This can help control the epidemic more quickly and accelerate the improvement of social conditions (2). Given the value of qualitative studies, it seems that such studies may help to better understand the staff subjective experiences regarding their stress and its consequences in working environment (13).

In this study, a qualitative research was designed to evaluate health care professionals' perception of stress during the COVID-19 pandemic in Iran.

## Materials and Methods

### Participants

Participants were recruited from staff members of different departments who were involved in the care of patients with COVID-19 infection in the largest teaching hospital of Tehran. Quota sampling was used to include physicians, nurses, and other paramedics. Participants were invited to be interviewed individually and sampling was continued until saturation. The time and place of the interview were scheduled keeping in view the convenience of the participants. At the end of sampling, 5 physicians, 10 nurses, and 5 other medical staff were included in the study. Of these, 11 were women and 9 were men. Before starting the interviews, the study was approved by the Ethics Committee of Tehran University of Medical Sciences (the Code of Ethics: IR.TUMS.VCR.REC.1399.044).

### Research Instruments

The 32-item consolidated criteria for reporting qualitative research (COREQ) checklist was used as the study tool in this qualitative research. This checklist is used to provide a clear and comprehensive report of qualitative studies and includes the components needed to evaluate in depth interviews, components related to characteristics of groups, research team, study design, data analysis, and reporting (14). Accordingly, approaches such as cross-cutting data and searching for patterns and themes, and other necessary components of qualitative studies were applied (15).

### Domain 1: Research Team and Reflexivity

#### Personal Characteristics

Two interviewers of the study were psychiatrists. The first interviewer was a woman and the fellow of psychosomatic medicine, and the second interviwer was a man and the professor of psychiatry. They had extensive experience in the field of psychosomatic disorders and worked at the largest medical hospital in Iran.

#### Relationship With Participants

The interviewers talked to health care professionals and a good relationship was established between them and the participants. Both of them had complete personal care equipment during the interview. Also, in these short conversations, which were conducted after identifying the individuals with the inclusion criteria, a discussion was held in a short session (10 min) about the objectives of the study and the reasons for doing the research. In these short sessions, before starting the main interview session, interviewers' concerns about the mental health of staff and their efforts to reduce their stress were discussed.

### Domain 2: Study Design

#### Theoretical Framework

The grounded theory was used to design the study (16).

#### Participants

Consecutive sampling was used for selection of participants (17). The total number of participants in the study was 20, of which 11 were female and 9 were male. Three people who had inclusion criteria were reluctant to participate. After selecting the final 20 cases, all of them participated in the study.

#### Setting

At the time of the interview, only health care professionals and interviewers were present. The interview room had a proper ventilation system, and both interviewers had full personal protective equipment. These interviews were conducted in the early days of the COVID-19 epidemic in Iran, with the aim of examining perceptions of stress among medical staff and assessing their needs in order to take effective measures to address the problems.

#### Data Collection

The questions, questionnaires, and guidelines were designed and modified before the study began. For the pilot phase of the study, prior to the main interview, a preliminary interview was conducted by each of the interviewers with individuals who had the inclusion criteria. All interviews were conducted in one session and were not repeated. Some important points were noted during the interview, but most of it was arranged in written form. Each session lasted about half an hour. The final text for each participant was re-submitted to medical staff for completion or correction.

### Domain 3. Analysis of Findings

#### Data Analysis

In this study, semi-structured interviews were implemented to give the participants freedom for creativity in their responses. The interview contained open-ended questions in different topics covering experiences of staff in caring patients with COVID-19 infection. Interview questions were developed based on a theoretical insight as well as professional team discussion. Individual appointments were made for conducting the interviews, which were held at the participants' workplace in a quiet room. All interviews were recorded using a smartphone. Before starting the interview, the participants were given information about the study and asked whether they wanted to participate utilizing an informed consent. After multiple reviews of the data, key concepts were selected by code formatting. Categories were derived based on the relationship between the codes and then formulated. The relationship between categories was demonstrated, and the thematic approach was used for content analysis (18, 19). According to Bengtsson's view, the purpose of content analysis is to organize and extract meaning from the data collected and to draw realistic conclusions (20). After removing irrelevant details, data gathering was continued until no new information was obtained. The correlation between the obtained data was evaluated in the inference stage. Finally, through process analysis, patterns that illustrated the results of the content analysis were identified and formulated. Data analysis was performed based on open and axial coding and after implementation of codes in MAXQDA software. The collected information was provided solely to the researchers to maintain confidentiality. In total, 20 interviews were conducted and 187 codes were obtained. These codes were also classified into 12 categories with 46 subcategories.

#### Reporting

The study report was elaborated by participants' quotations. They were written in accordance with the themes, categories, and subcategories obtained. The consistency between the data presented and the findings was observed. The main themes were clearly presented in the findings, and various themes and sub-themes were discussed.

## Results

The total number of participants in this study was 20 individuals [11 females (55%) and 9 males (45%)]. Among them, 5 were physicians, 10 were nurses, and 5 were other paramedics. The average age of the participants in the study was 35.5 years (SD = 8.46). The minimum age was 23 and the maximum age was 51 years. Nine participants worked in the intensive care unit (ICU), five in the infectious diseases ward, three in the respiratory diseases ward, and three in the internal medicine ward. The average work experience of the staff was 10.5 years (SD = 7.45), which included at least 1 year and a maximum of 26 years.

Most of the participants reported experiencing stress in the face of COVID-19 epidemic working conditions. They were very anxious, deeply affected by the death of their patients, and worried about the transmission of the disease to their families. During the interviews, they talked about stress manifestations, awareness, and understanding of different aspects of COVID-19 and crisis management. Also, the suggested solutions for overcoming the existing situation were discussed.

In this study, 4 themes were found; they are uncertainty accompanied by the pandemic of COVID-19 (explains the experience of stress), shortcomings in preparation for crisis management (explains the experience of stress), manifestations of stress, and mental health needs. Each theme consisted of several categories and sub-categories (Tables 1, 2).

**Table 1:** Uncertainty and crisis management related to perception of stress by medical staff during COVID-19 epidemic.

**Themes**	**Categories**	**Subcategories**	**Segments**
“Uncertainty” accompanied by the COVID-19 pandemic (Explains the experience of stress)	Predisposing factors	The inconsistency in medical information	“One doctor expresses an opinion, while another doctor says a contradictory opinion about the disease”
		The obscurity of the disease	“In the early phase, we were under stress because we had no information and no one guided us”
		The likelihood of transmission to the family	“We still have concerns of our family being infected. We should not meet our parents because they are old”
		Unforeseen exposure to infectious droplets	“We are concerned about unexpected exposures and the ways of protection”
		The likelihood of getting infected	“I am very worried about getting infected due to my asthma”
		Being shocked in the early days	“The first week was very stressful. There was no preparation at all; the medical system denied the health crisis”
		Resource allocation	“Making decision is difficult on several issues such as concerns about patient's priorities for critical care and ICU and the necessity to retain employees who are reluctant to stay in the work place”
	Protective factors	Visiting patients who have recovered	“It can be motivating to see patients who have recovered”
		Believing in better prognosis in youth	“Most of us are young and therefore we are at lower risk”
		Paying attention to the positive aspects of COVID-19	“Human beings are members of a whole, in creation of one essence and soul”
		Hoping not to get infected	“I'm not worried because most people are asymptomatic; I think I'm not infected”
		Self- reassurance due to personal care	“I'm a medical staff and more aware about personal hygiene, so it will be easier for me to handle the problems; therefore, I will not be infected”
		Reducing the stress by observing the downward trend of the disease	“When we see that the process of hospitalization and admission of patients is improved, we are less concerned”
Shortcomings in preparation for crisis management	Predisposing factors	Shortage of personal protective equipment (PPE)	“If there are only masks and gloves, there would be a lot of stress, but it would be easier if I have complete PPE”
		Personal problems	“My sister-in-law was infected and only I knew, and now I have to take care of her along with my work”
		Management factors	“There was the lack of coordination. A more coherent and orderly plan should have been provided. That is, tasks had to be identified, and division of labor should be done”
	Protective factors	Resource management	“If the cost spent on treating infected staff, sick leave, and on a possible CT scan was allocated to PPE, both the stress and the suffering of colleagues would decrease”
		Spiritual factors	“We put our trust in God”
		Altruism	“We tried to come up with the idea that we were serving our people”

**Table 2:** Manifestations of stress and mental health needs related to perception of stress by medical staff during COVID-19 epidemic.

Manifestations of stress	Anxiety	Negative impact of stress on personal life	“Stress had a disruptive effect on my daily and personal life”
		Mental symptoms of anxiety	“I'm very worried”
		Avoiding work	“Two or three persons didn't work and left the ward and went out”
		Somatic symptoms	“I had headache and palpitation”
	Depression		“We feel low. We are not happy and do not laugh. During morning sessions, we are mostly tired”
	Anger		“We got into a fight; one colleague got angry and left work”
	Feelings of guilt	Transmission of infection to the family	“I feel guilty if I transmit the disease to my family; I feel guilty about my family”
		Patient death	“They always complained about the services and care. The patient could have survived”
	Hopelessness		“Most colleagues are frustrated and don't know when COVID-19 is going to end”
	Feeling exhausted		“The stress of dealing with patients, the stress of family, and the fact that we can be carriers all make us unsettled”
Mental health needs	Crisis management	Careful monitoring and control of infection	“They are very carefree, especially in providing guards and services and monitoring and controlling infection”
		Obtaining public and charitable donations	“The friends of one of our colleagues donated money. We provided financial assistance to our service staff”
		Choosing the right encouragement based on one's work experience	“Financial incentives may not be very important to experienced staff, but it does motivate the younger”
		Providing adequate protection equipment	“At least, staff should be provided with personal protective equipment. We provide N95 masks for three or four shifts. This is not correct”
		Appropriate distribution of equipment	“The allocation of the equipment and its distribution among the colleagues must be done to benefit everyone”
		Legal support for physicians and nurses	“Tariffs for nursing services have been in place for 10 years but have not yet been implemented. This should be verified and the budget should be allocated to it”
		Appreciation	Anything that makes the staff happy is favorable. So, appreciation is effective and its effect would be synergized if they receive appreciation from hospital and authorities outside; such effect from outside is more important”
		Increasing the number of staff required per shift	“They need to increase the number of personnel per shift”
		Compensatory leave	“They have to say you can go on leave for 2 extra weeks”
		Reducing the number of shifts	“Our encounters with patients should be reduced; that is, the number of our shifts should be decreased”
		Reducing job discrimination	“Many of our colleagues are now leaving because they are afraid of COVID-19. Of course, there must be a difference between us and them. We agreed to come and work”
		Planning for unexpected events	“Planning and management must be done for future incidence”
		Distribution of responsibilities	“Many other wards of the hospital were closed; if they were working, we could get help from them to reduce the long shits duration”
		Reducing family exposure	“Even my sister-in-law is undergoing chemotherapy; my wife has not been able to see her sister for a month because of my job. After all, my children are carriers”
	Stress management	Stress management training	“We need to learn how to lessen psychological and emotional strains when we go home or in the ward”
		The need for availability of a psychologist / psychiatrist in the ward	“It would be better to talk in the group meetings we have, such as the morning report sessions, about what to do with our anxiety”
		Improving the quality of communication between professors and residents	“If, instead of the representatives, the residents themselves speak directly to the professors, the staff will be more encouraged”
		Participation of professors alongside residents	“Residents realized that the situation was so critical that the professors themselves came to work directly with the patients, and as a result, the collaboration of residents gradually improved”
		Arranging quarantine for staff	“To reduce the risk of transmission, we will be quarantined in the hospital for 10 days, then another group will come”
		Producing educational content for family members	“If they are trained through a program like a video clip, their negative perception would decrease”
		Facilitating access to a psychologist / psychiatrist	“We really need a psychologist because a lot of residents have major problems. In a crisis, they really lose the power of reasoning due to stress. If there would be a place to help, it will be effective”

## Discussion

In this qualitative study, by emphasizing on two main dimensions of the disease through raising “how” and “why” questions (13), an attempt was made to gain insight into how stress is experienced and how it is manifested among health care staff who are involved with COVID-19 patients. In our study, health care professionals experienced stress in several ways as they enumerated anxiety, depression, anger, feelings of guilt, and a sense of hopelessness and burned out as the main symptoms of the stress during providing care for patients.

In a multicenter study about health care workers' (HCWs) mental health status in Iran during COVID-19 pandemic, depression was negatively associated with most quality of life domains, yet, social support was positively correlated with physical function, energy, and emotional well-being (21). In another multicenter study in Iran, the prevalence of posttraumatic stress disorder among HCWs was reported to be more than one third of the whole population (22). Also, in another multicenter study in Iran, about 40% of HCWs had moderate to severe anxiety which was more severe in women, nurses, and younger people (23).

Thus, it seems that the experience of stress among HCWs in Iran, in terms of above categories, is the dominant image of their mental state during COVID-19 pandemic so far. The perceived strong sense of danger associated with feelings of ambiguity regarding the pandemic and the unsolved mysteries created by the virus has been the experience of another group of HCWs in Iran during the recent pandemic (24).

In our study, the uncertainty accompanied by the COVID-19 epidemic in conjunction with inconsistent medical information and obscurity of the disease could have been the main causes of stress among staff and other factors like downward trend in disease could reduce or even eliminate their stress.

In a study conducted at a teaching hospital following the outbreak of severe acute respiratory syndrome (SARS) in 2003, the obscurity of the disease, was one of the important factors for fear and stress in caregivers similar to our study (2). In general, uncertainty is a strong stressor and there are, of course, individual differences in its impacts (25). In our study, this feature was also reported by a group of staff as a stressor associated with COVID-19.

Mental symptoms of anxiety, such as worry and life-threatening stress, were seen among health personnel in our study. A similar experience has been reported with staff at one of the COVID-19 care centers in Wuhan, China (7). Cai et al. examined the psychological status of health care providers during the outbreak of the COVID-19 and reported increased stress among them (26).

Psychological stress can be identified by its acute or chronic somatic symptoms (27). Headaches and palpitation were among the manifestations of stress among our participants. In addition, they were psychologically affected by hearing the deaths of COVID-19 patients. Among the stressful manifestations of the SARS epidemic, concerns about the transmission of the disease to the family were the typical ones. Anxiety and anger were other manifestations of health staff which are the same as the feeling of our health care providers (2).

In a study in Wuhan, Zhu et al. reported unprecedented psychological stress in the medical staff dealing with COVID-19 patients (11). The staff's concern about the transmission of the disease to themselves and their families, as a stressful experience, and the unknown nature of the disease as a stressor in their study is similar to our results. The unpredictability of COVID-19 disease has also been emphasized as an important factor in causing stress among individuals in previous research (12).

Conditions such as insufficient information in the early stages of COVID-19 spread and difficulty in deciding on new treatments have been highlighted as stressors in some other studies (5–7). Also in a recent research in Iran, nearly half of HCWs (nurses) had good knowledge about the disease, its transmission, and related treatment methods (28). It seems that increasing education in this field and transferring national and international scientific experiences is one of the beneficial approaches to fill this educational gap. Also, due to the pandemic conditions, the use of online training programs, expanding virtual training options, and identifying and introducing reliable and accurate sources of information may be helpful approaches in overcoming the challenges created by COVID-19 infection.

Lack of protective equipment was one of the causes of stress in the management of COVID-19 disease in our study. Unfortunately, similar conditions and limitations were observed in previous research as well (7). In the field of crisis management, the need for adequate equipment has been the chief concern of our medical staff. Zhu et al. have cited this as a contributing factor in reducing stress, anxiety, and depression (11).

Considering the shortcomings in crisis management, the main stressor in our study was lack of adequate protective equipment and the major stress reliever was resource management in health care staff. Crisis management through careful monitoring and control of infection, and provision of adequate protective equipment in tandem with stress management through availability of a psychologist/psychiatrist are mental health measures to reduce stress in caregivers.

Relative shortage of care equipment, mentioned in our study, can be attributed to the economic problems in Iran (29). This issue, along with the unfavorable crisis management conditions, has exacerbated the situation in monitoring and controlling the affected cases. It seems that the existing restrictions on protective equipment, along with other economic problems in Iran, including concerns about the living conditions of people in the community have complicated the situation. However, the effect of other social, cultural, and political factors cannot be neglected (30) and the synergistic influences of several measures such as financial and human resource management, support measures, and crisis management seem to have a role in improving the situation (31).

The main prerequisite in crisis management as part of the health care staff's needs is strict monitoring and control of infection. This requirement has been emphasized in some other studies (5). Similar to some studies of COVID-19, inadequate education on infection control has been associated with higher levels of anxiety and depression (32).

Our study showed that health care professionals have reported the value of personnel incentives, feelings of gratitude, proper distribution of equipment, and the reduction of occupational discrimination. In a qualitative study, Olofsson et al. pointed out that nursing is a risky occupation due to encountering with stressful diseases (33). They argued that failure to respond appropriately to their demands could induce feelings of hopelessness and powerlessness.

The burnt-out feeling experienced by emergency medicine assistants and the need for psychologic support for appropriate coping with occupational problems were emphasized in previous research (34).

One of the protective factors for stress in our study was the influence spiritual beliefs and altruism among medical staff. In some studies, the relationship between spiritual beliefs and altruism with better mental health situation has been reported among health care providers. McKee et al. reported that people with stronger spiritual beliefs showed greater resistance to the negative effects of stress at work (35). Consistent with several studies, spirituality and faith have been reported as a source for coping with adverse health conditions (36). In a review study in Iran, regarding the role of spirituality and religion in dealing with crisis, such an approach has been introduced as a way to help create mental relaxation. The study also emphasizes resorting to such beliefs and feelings in the face of the recent pandemic crisis (37).

## Conclusion

Based on the findings of the current study, it is important to identify and evaluate the mental health needs of healthcare professionals. To reduce stress among health staff at COVID-19 care centers, it seems that simultaneous improvement in equipment and crisis management are optimal strategies. Providing facilities for staff stress management seems to be effective as well. However, it seems that in order to help improve the mental health of HCWs, further studies should be conducted in other fields related to mental health.

One of the findings of our study was the lack of knowledge about the baseline mental health status of HCWs. Also, the role of various factors such as economic factors in shaping the perception of stress among HCWs was indicated. A more detailed multicenter research evaluating the impact of these factors is required to be performed in the future as our study was conducted in a single center.

## Data Availability Statement

The original contributions presented in the study are included in the article/supplementary material, further inquiries can be directed to the corresponding author/s.

## Ethics Statement

The studies involving human participants were reviewed and approved by Ethical Committee of Tehran University of Medical Sciences (the Code of Ethics: IR.TUMS.VCR.REC.1399.044). The patients/participants provided their written informed consent to participate in this study.

## Author Contributions

All authors: conception and design of the study, searching for articles, and writing the final manuscript.

## Funding

This study was supported by a grant from Tehran University of Medical Sciences (Grant Number: 99-1-101-47250).

## Conflict of Interest

The authors declare that the research was conducted in the absence of any commercial or financial relationships that could be construed as a potential conflict of interest.

## Publisher's Note

All claims expressed in this article are solely those of the authors and do not necessarily represent those of their affiliated organizations, or those of the publisher, the editors and the reviewers. Any product that may be evaluated in this article, or claim that may be made by its manufacturer, is not guaranteed or endorsed by the publisher.
